# Physical rehabilitation interventions in the intensive care unit: a scoping review of 117 studies

**DOI:** 10.1186/s40560-018-0349-x

**Published:** 2018-12-07

**Authors:** Julie C. Reid, Janelle Unger, Devin McCaskell, Laura Childerhose, David J. Zorko, Michelle E. Kho

**Affiliations:** 1 Faculty of Health Sciences, School of Rehabilitation Science, McMaster University, Institute of Applied Health Sciences, Room 403, 1400 Main Street West, Hamilton, ON L8S 1C7 Canada; 2 Rehabilitation Sciences Institute, University of Toronto, Rehabilitation Sciences Building, 500 University Avenue, Suite 160, Toronto, ON M5G 1V7 Canada; 3 Department of Physiotherapy, St. Joseph’s Healthcare Hamilton, 50 Charlton Avenue East, Hamilton, ON L8N 4A6 Canada; 4 Department of Pediatrics, McMaster University, 1280 Main Street West, Hamilton, ON L8S 4K1 Canada

**Keywords:** Rehabilitation, Critical care, Critical illness, Respiration, artificial, Early ambulation

## Abstract

**Background:**

Physical rehabilitation (PR) interventions in the intensive care unit (ICU) can improve patients’ functional outcomes, yet systematic reviews identified discordant effects and poor reporting. We conducted a scoping review to determine the extent of ICU PR interventions and how they were reported and measured.

**Methods:**

We searched five databases from inception to December 2016 for prospective studies evaluating adult ICU PR interventions. Two independent reviewers screened titles, abstracts, and full texts for inclusion. We assessed completeness of reporting using the Consolidated Standards of Reporting Trials, Strengthening the Reporting of Observational Studies in Epidemiology, or Standards for Quality Improvement Reporting Excellence guidelines, as appropriate. For planned PR interventions, we evaluated reporting with the Consensus on Exercise Reporting Template (CERT) and assessed intervention and control groups separately. We calculated completeness of reporting scores for each study; scores represented the proportion of reported items. We compared reporting between groups using Kruskal-Wallis with Bonferroni corrections and *t* tests, *α* = 0.05.

**Results:**

We screened 61,774 unique citations, reviewed 1429 full-text publications, and included 117: 39 randomized trials, 30 case series, 9 two-group comparison, 14 before-after, and 25 cohort. Interventions included neuromuscular electrical stimulation (NMES) (14.5%), passive/active exercises (15.4%), cycling (6.8%), progressive mobility (32.5%), and multicomponent (29.9%). The median (first,third quartiles) study reporting score was 75.9% (62.5, 86.7) with no significant differences between reporting guidelines. Of 87 planned intervention studies, the median CERT score was 55.6%(44.7,75.0); cycling had the highest (85.0%(62.2,93.8)), and NMES and multicomponent the lowest (50.0% (39.5, 70.3) and 50.0% (41.5, 58.8), respectively) scores. Authors reported intervention groups better than controls (*p* < 0.001).

**Conclusions:**

We identified important reporting deficiencies in ICU PR interventions, limiting clinical implementation and future trial development.

**Electronic supplementary material:**

The online version of this article (10.1186/s40560-018-0349-x) contains supplementary material, which is available to authorized users.

## Introduction

Adult survivors of critical illness can face profound, long-term functional impairments [1, 2]. Physical rehabilitation (PR) interventions can help improve functional outcomes [3], and minimizing the morbidity associated with critical illness through early intervention in a patient’s intensive care unit (ICU) stay is of great interest [4]. However, recent randomized clinical trials (RCTs) reported conflicting results on the effectiveness of ICU PR to improve patient outcomes [5–9]. While these discordant results could be due to differences in interventions, recent systematic reviews [3, 10] and a review of reviews [11] highlighted inconsistencies in PR study reporting, which impairs understanding the types and amounts of PR provided in these trials [3]. While systematic reviews evaluate a narrow range of studies to answer focused questions of effectiveness, scoping reviews have a broader mandate to examine the range and extent of research activity in a field [12].

Given the reporting deficiencies identified by systematic reviews, the types of PR interventions received by patients in the ICU are not clear. To address this gap, we conducted a scoping review of prospective studies to better understand the types and amounts of PR studied in ICU patients. Our research question was as follows: What is the extent of the original prospective research for PR interventions in critically ill, mechanically ventilated (MV) patients and how is PR reported and measured? Some of the results of this study have been previously reported in the form of an abstract [13].

## Methods

We followed a standardized scoping review methodology [12, 14]. We consulted a health research librarian, identified relevant databases, and developed and piloted the search strategy (Additional file 1: Table S1). A research librarian peer-reviewed the search strategy [15]. We searched the following databases from inception to December 31, 2016: OVID Medline, Cumulative Index to Nursing and Allied Health Literature (CINAHL), Allied and Complementary Medicine Database (AMED), Excerpta Medica database (EMBASE), Physiotherapy Evidence Database (PEDro), and authors’ personal files.

### Inclusion/exclusion criteria

We included *Population*—adult ICU patients receiving MV; *Interventions*—PR initiated in the ICU (e.g., neuromuscular electrical stimulation (NMES), passive or active exercises, strengthening exercises, sitting, cycling, progressive mobility, or any combination thereof); *Comparator* and *Outcomes*—any or none; *Studies*—prospective original research (e.g., RCTs (inter-patient, within-patient, crossover), two-group comparison studies, case series, before-after, cohort studies). We excluded studies with interventions started outside the ICU, chest physiotherapy or other respiratory-type interventions, studies of outcome measures, and surveys of practice. We also excluded non-English language studies, grey literature, review articles, retrospective studies, and qualitative research.

We imported all citations into Covidence (2015 Veritas Health Innovation, Melbourne, Australia) and conducted calibration exercises to optimize reviewer agreement. Two independent reviewers assessed all titles, abstracts, and full-text citations; a two-reviewer agreement was required to advance a citation through the review process. Disagreements were resolved by consensus, using a third reviewer where necessary.

### Data collection

We developed electronic data collection forms. One reviewer extracted data from the main trial publication, related publications, and any referenced published protocols or additional files. A second reviewer independently reviewed all publications and extracted data for accuracy. We extracted study characteristics (clinical setting, severity of illness scale, study design, sample size, intervention, outcomes), patient demographics (age, sex, admission diagnosis), and intervention details (intervention types, amounts, start times, duration). We organized interventions into single- and multicomponent categories (Additional file 1: Table S2).

We assessed overall study and intervention reporting. In duplicate, we assessed the quality of study reporting by design: Consolidated Standards of Reporting Trials (CONSORT) [16] for RCTs and two-group comparison studies, Strengthening the Reporting of Observational Studies in Epidemiology (STROBE) [17] for cohort studies and case series, and Standards for Quality Improvement Reporting Excellence (SQUIRE) [18] for quality improvement studies and before-after trials. Current study reporting tools (e.g., CONSORT, STROBE, SQUIRE) provide important direction for overall study reporting; however, they do not provide sufficient guidance for reporting of complex interventions such as PR.

Recently, adjunct tools such as the Consensus on Exercise Reporting Template (CERT) provide direction for explicit exercise intervention reporting [19]. CERT evaluates a core set of items essential for exercise intervention replication, and while it was not developed specifically for ICU, it is reported to be equally applicable across all health and disease states [19]. For studies of planned PR interventions, we assessed reporting with CERT [19]. We chose CERT versus other tools such as the Template for Intervention Description and Replication (TIDieR) checklist, because CERT more explicitly evaluates essential items for replication such as dosage, supervision requirements, and tailoring requirements [19]. Where applicable, we evaluated intervention and control groups separately. To evaluate dosage (CERT item 13), we assessed frequency, intensity, timing, and duration discretely rather than as a single item. We excluded studies that did not evaluate a planned PR intervention from CERT assessment (e.g., mandatory mobility orders [20]).

### Analysis

We visually inspected data using box plots and assessed normality using the Shapiro-Wilk test. We summarized descriptive data using counts and percentages for categorical variables, means and standard deviations for continuous variables, or medians and first and third quartiles if data were skewed. We collated study content by intervention category. For each study, we calculated the completeness of reporting as the proportion of the reported items divided by the total items for the corresponding reporting guideline (e.g., CONSORT, STROBE, SQUIRE, or CERT), minus items not applicable to the study. To evaluate *study reporting*, we grouped the studies by intervention category and reporting guideline. To evaluate *intervention reporting*, we grouped studies by intervention category, reporting guideline, and intervention/control group. Additional file 1: Table S3 outlines decision rules for assessment denominators. We classified the quality of reporting using ≥ 70% and ≤ 50% for adequate and poor, respectively, [21, 22] and scores between 50 and 70% as moderate.

We compared the reporting scores across intervention categories and reporting guidelines. Due to skewed distributions and small sample sizes for some groups, we used the Kruskal-Wallis test [23] then conducted pairwise comparisons with Bonferroni corrections (*α*/number of groups) to identify specific differences (intervention category *α* = 0.05/5 = 0.01; reporting guideline *α* = 0.05/3 = 0.0167). To compare intervention and control group reporting, we used *t* tests. We used two-tailed tests with non-directional hypotheses with a critical *α* = 0.05. All analyses were carried out using Stata (v. 14.2, College Station, Texas: StataCorp LP).

## Results

**Fig. 1:**
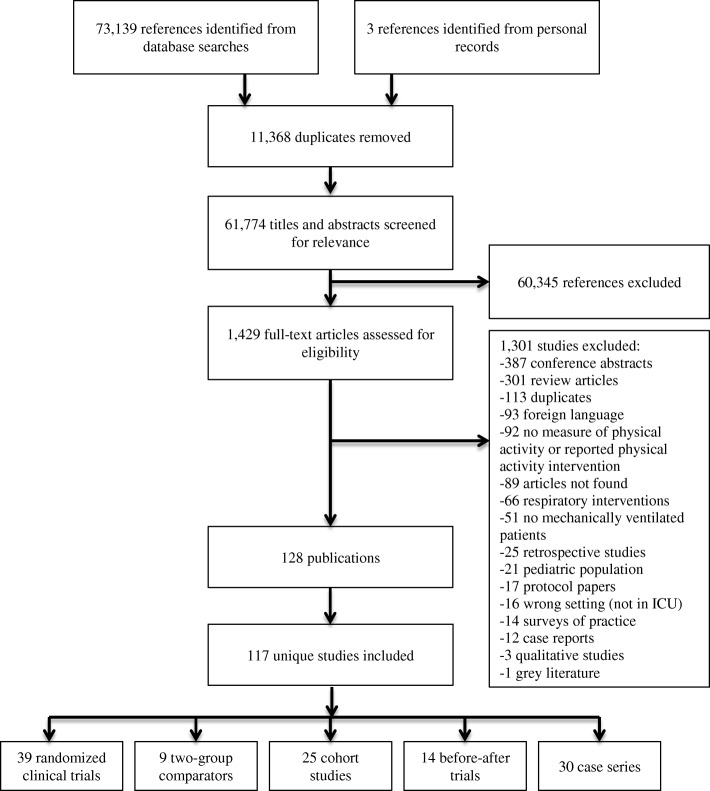
PRISMA flow diagram of included studies

**Fig. 2:**
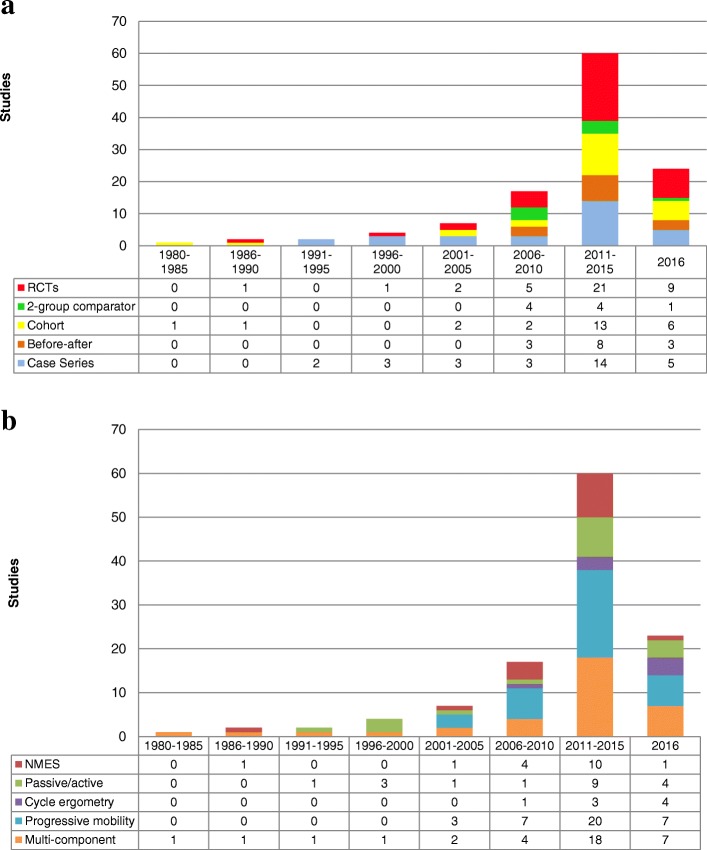
**a** Distribution of ICU physical rehabilitation study designs and **b** intervention types over 32 years

We identified 73,142 potentially eligible citations, 61,774 unique citations after de-duplication, and 117 unique studies met inclusion criteria (Fig. 1). Table 1 summarizes the characteristics of included studies and the demographics of included patients. Figure 2a, b shows the distribution of study designs and interventions over time. Seventy-two percent (*n* = 84) of adult PR studies emerged after 2010, 41 (35.0%) occurred in the USA and 17 (14.5%) in Australia. The majority of studies were single-center (107 (91.5%)), conducted in medical/surgical (22 (18.8%)), mixed (22 (18.8%)), or medical (20 (17.1%)) ICUs. The 117 studies enrolled 17,915 patients, 50.7% (9078) male, with a median (first, third quartiles) age of 60 (55, 64). The most frequent admission diagnosis categories were respiratory-related (2737 (15.3%)) followed by neurological (2074 (11.6%)) and post-surgical (1891 (10.6%)). The median sample size was 57 (25, 112). RCTs accounted for 33.3% (*n* = 39) of all studies, of which 76.9% (*n* = 30) were published after 2010. Of the 39 RCTs, 32 (82.1%) randomized between patients, 7 (17.9%) within patients, and they enrolled a median of 46 (28, 87) patients.

**Table 1:** Characteristics of 117 included studies and demographics of included patients

Study characteristics	
Countries, *n* (%)	
USA	41 (35)
Australia	17 (15)
Brazil	9 (8)
Belgium	8 (7)
Italy	6 (5)
UK	5 (4)
Taiwan	4 (3)
Greece	3 (3)
Others^*a*^	24 (21)
Number of centers, *n* studies (%)	
Single-center	107 (91)
Multi-center	10 (9)
Median number of centers (first, third quartiles)	5 (5, 14)
ICU type, *n* (%)	
Medical/surgical	22 (19)
Mixed (medical, surgical, cardiovascular, neurological)	22 (19)
Medical	20 (17)
Neurological	15 (13)
Respiratory	9 (8)
Surgical	9 (8)
Cardiovascular	3 (3)
Not reported	17 (15)
Reported Severity of Illness Scale, *n* studies (%)	
APACHE II	58 (50)
APACHE III	7 (6)
SAPS II	3 (3)
SOFA	3 (3)
Others^*c*^	7 (6)
Not reported	38 (32)
Study design, *n* (%)	
Randomized clinical trial^b^	39 (33)
Case series	30 (26)
Cohort	25 (21)
Before-after	14 (12)
Two-group comparison	9 (8)
Sample size (enrolled), median (first, third quartiles)	
Randomized clinical trial^b^	46 (25, 87)
Case series	23 (15, 60)
Cohort	101 (43, 246)
Before-after	141 (80, 582)
Two-group comparison	59 (24, 193)
Overall	57 (25, 112)
Intervention type, *n* (% of all types)	
Progressive mobility	38 (32)
Multicomponent	35 (30)
Passive or active exercise alone	18 (15)
Neuromuscular electrical stimulation	17 (15)
Cycling	8 (7)
Unable to classify	1 (1)
Patient demographics	
Age (years) median (first, third quartiles)	59.4 (55.0, 63.9)
Sex (male, *n* patients (%))	9078 (51)
Admission Diagnosis, *n* patients (% of total)	
Respiratory	2737 (15)
Neurological	2074 (12)
Post-surgical	1891 (11)
Medical	1464 (8)
Cardiovascular	1094 (6)
Sepsis/infection	1024 (6)
Unspecified	4218 (24)
Others^*d*^	504 (3)
Not reported, *n* studies	15

*APACHE II* Acute Physiology and Chronic Health Evaluation II, a 13-item instrument with scores from 0 to 71, higher scores representing more severe illness; *APACHE III* Acute Physiology and Chronic Health Evaluation III, a prognostic scale with scores from 0 to 299, higher scores indicating a poorer prognosis; *SAPS II* Simplified Acute Physiology Score II, a 17-item scale with scores from 0 to 163, higher scores representing increased risk of hospital mortality; *SOFA* Sequential Organ Failure Assessment, a 6-item scale to predict mortality with scores from 6 to 24, higher scores indicate poorer prognosis

^a^Other includes China (*n* = 2), Austria (*n* = 2), Turkey (*n* = 2), Germany (*n* = 1), France (*n* = 2), Sweden (*n* = 2), 1 study each from Israel, South Korea, Egypt, South Africa, Japan, Kuwait, Argentina, Denmark, Canada, Zimbabwe, Spain, India, Switzerland

^b^Randomized controlled trial includes randomized cross-over designs (*n* = 3) and within-patient randomized designs (*n* = 4)

^c^Other included Glasgow Coma Scale (*n* = 3), Simplified Acute Physiology Score III (*n* = 2), Brunnstrom (*n* = 1), Injury Severity Score (*n* = 1), Braden Scale (*n* = 1)

^d^Other includes trauma (*n* = 397, 2.2%), oncology (*n* = 41, 0.2%), transplants (*n* = 43, 0.2%), extracorporeal membrane oxygenation or left ventricular assist device support (*n* = 9, 0.1%), musculoskeletal (*n* = 11, 0.1%), burns (*n* = 3, 0.02%)

### Interventions

Of 117 studies, we were unable to classify 1 study into an intervention category due to limited reporting [24]. Of the remaining 116, single interventions accounted for 69.8% (*n* = 81). The 2 most common interventions were progressive mobility (38 (32.8%)) and multicomponent (35 (30.2%)). Below, we describe interventions organized from single to multicomponent and by participant engagement (passive to active). Additional file 1: Table S4 summarizes the studies.

Of 81 single interventions, NMES occurred in 17 (21.0%), with 14 of these (82.3%) being RCTs. Of the NMES studies, 16 (94.2%) studies targeted the lower extremity muscles, 1 also included biceps [25], and 1 studied accessory respiratory muscles [26]. Start times for the interventions varied from 1 [27, 28] up to 4.6 days [29] after ICU admission with intervention duration varying from a single session [30] up to 4 weeks after initiation [31]. Three studies did not report information on the intervention start time [26, 28, 30].

The most common single intervention was progressive mobility (38 (46.9%) studies, including 5/38 (13.2%) RCTs). Progressive mobility typically began with passive or active exercises progressing to ambulation. Interventions started as early as the day of ICU admission [32] and some lasted throughout the hospital stay [33]. Eight studies did not report start time [34–41], and 12 did not report intervention duration [32, 34–36, 38–45].

The remaining 26 (32.1%) single intervention studies were passive/active exercises (18 (22.2%)) and cycling (8 (9.9%)). One study evaluated passive intervention and cycling intervention, both compared to a control group [46]. Eight of these 26 (30.8%) were RCTs (5 passive/active, 3 cycling). Passive/active exercise studies included sitting (*n* = 3), passive range of motion (*n* = 7), active-assisted or active range of motion (*n* = 2), positioning (*n* = 4), and tilt table (*n* = 3). All cycling studies used leg ergometers. Interventions started between 2 [47] and 14 days [5] after ICU admission, and intervention duration varied from 1 day [48, 49], 15 sessions [50], the duration of the ICU stay [5, 51], and up to 4 weeks [52]. Six studies did not report start time [51, 53–57].

Multicomponent interventions comprised 30.2% (*n* = 35) of all studies, with 12 (34.3%) RCTs. These studies included a median (first, third quartiles) of 3 (2, 4) components, with a minimum of 2 and maximum of 5. We could not discern the number of components in 1 study because the admission diagnosis determined the intervention algorithm [58]. Components included NMES (*n* = 3), passive/active exercises (*n* = 21), cycling (*n* = 9), and progressive mobility (*n* = 27). Other interventions included respiratory techniques (e.g., breathing and coughing exercises, manual lung hyperinflation, suctioning, and postural drainage (*n* = 15)), muscle strengthening programs (*n* = 11; skeletal = 8, respiratory = 3), activities of daily living training (*n* = 3), education (*n* = 3), cognitive training (*n* = 1), interactive videogame programs (*n* = 1), proprioceptive neuromuscular facilitation techniques (*n* = 1), global kinesiotherapy (*n* = 1), and multisensory stimulation (*n* = 1). Interventions started from 1 [7, 59] to 15 days post-ICU admission [60], with reported overall duration from a minimum 10 sessions [61], some extending to the outpatient setting [8, 59, 62]. Intervention start times and duration were not reported in 11 [61, 63–72] and 7 studies [60, 66–68, 72–74], respectively.

Additional file 1: Figure S1a shows all 20 single component inter-patient RCTs by intervention category, start time, and intervention duration. Additional file 1: Figure S1b shows all 12 multicomponent inter-patient RCTs by intervention category, start time, and intervention duration.

### Quality of reporting

#### Overall study reporting

Table 2 summarizes reporting by intervention category (*n* = 116 classified studies) and guideline (*n* = 117 studies). The median (first, third quartiles) score was 75.9% (62.5, 86.7). Progressive mobility studies had the highest reporting (79.3% (70.4, 87.2)) and NMES studies, the lowest (66.7% (54.8, 83.3)). There were no significant differences in study reporting across intervention categories or reporting guideline.

**Table 2:** Summary of reporting scores. Scores reported as median proportion (first, third quartiles)

(i) Study design reporting (*N* = 117)						
	NMES (*n* = 17)	Passive/active (*n* = 18)	Cycling (*n* = 8)	Progressive mobility (*n* = 38)	Multi-component (*n* = 35)	Overall (*n* = 117)
CONSORT (*n* = 48)	64.59 (53.99, 81.36)	75.86 (65.52, 80.00)	80.00 (75.76, 87.88)	75.81 (48.39, 96.97)	79.07 (54.70, 87.10)	74.98 (54.84, 87.10)
STROBE (*n* = 55)*	83.33	64.24 (59.26, 77.78)	85.19 (70.00, 89.29)	77.99 (73.22, 86.44)	73.24 (64.29, 76.92)	75.00 (64.29, 86.21)*
SQUIRE (*n* = 14)	–	90.00	–	82.50 (70.00, 87.34)	85.00	82.50 (75.00, 87.5)
Overall (*n* = 117)*	66.67 (54.84, 83.33)	73.76 (62.07, 80.00)	82.60 (72.88, 88.58)	79.31 (70.37, 87.18)	74.19 (55.56, 86.67)	75.86 (62.50, 86.67)*
(ii) Overall CERT reporting (*N* = 87)						
	NMES (*n* = 17)	Passive/active (*n* = 16)	Cycling (*n* = 8)	Progressive mobility (*n* = 22)	Multi-component (*n* = 24)	Overall (*n* = 87)
CONSORT (*n* = 48)	47.98 (39.38, 61.53)	70.59 (57.14, 75.00)	58.54 (48.01, 65.79)	53.95 (45.24, 62.50)	48.69 (36.12, 55.26)	51.99 (41.57, 64.56)
STROBE (*n* = 30)	94.44	63.75 (54.79, 74.38)	87.5 (86.67, 100)	57.89 (52.63, 81.25)	58.82 (42.86, 77.78)	68.59 (55.56, 83.33)^a^
SQUIRE (*n* = 9)	–	90.00	–	47.37 (37.5, 60.53)	42.86	47.37 (42.86, 60.53)
Overall (*n* = 87)	50.00 (39.47, 70.27)	66.88 (56.70, 77.50)^b^	85.00 (62.16, 93.75)^c, d, e^	54.10 (47.37, 65.00)	50.00 (41.79, 59.17)	55.56 (44.74, 75.00)
(iii) Intervention and control group reporting with CERT (*N* = 57)						
	NMES (*n* = 16)	Passive/active (*n* = 8)	Cycling (*n* = 3)	Progressive mobility (*n* = 13)	Multi-component (*n* = 17)	Overall (*n* = 57)
Intervention (*n* = 57)	65.48 (61.11, 72.95)^f^	72.80 (68.34, 80.10)	68.42 (46.32, 76.19)	73.68 (57.89, 80.00)^g^	63.16 (47.37, 80.95)^h^	68.42 (55.56, 80.00)^i^
Control (*n* = 57)	26.90 (12.70, 63.16)	69.51 (59.41, 78.13)	42.11 (40.00, 63.16)	30.00 (10.53, 47.37)	36.84 (26.32, 47.37)	38.89 (21.05, 55.00)

Summary of reporting scores by (i) study reporting by reporting guideline and intervention categories, (ii) intervention reporting by reporting guideline and intervention categories, (iii) intervention and control group reporting by intervention categories. *One cohort study could not be classified into an intervention category; its reporting data is included in the overall STROBE reporting and overall study reporting scores, respectively

^a^Studies assessed with STROBE had significantly better CERT reporting compared to those assessed with CONSORT, *p* = 0.014

^b^Sitting/passive/active interventions reported significantly better than multicomponent interventions, *p* = 0.009

^c, d, e^Cycling interventions reported significantly better than NMES interventions, progressive mobility interventions, and multicomponent interventions, *p* = 0.012, *p* = 0.010, *p* = 0.002, respectively

^f^NMES intervention groups reported significantly better than control groups, *p* < 0.001

^g^Progressive mobility intervention groups reported significantly better than control groups, *p* < 0.001

^h^Multicomponent intervention groups reported significantly better than control groups, *p* < 0.001

^i^Across all intervention categories, intervention groups reported significantly better than control groups (*p* < 0.001)

#### Intervention reporting

We included 87 (74.4%) studies in our CERT analysis; the overall median reporting score was 55.6% (44.7, 75.0). By intervention category, cycling was highest at 85.0% (62.2, 93.8), while NMES and multicomponent studies were lowest, with scores of 50.0% (39.5, 70.3) and 50.0% (41.8, 59.2), respectively. Cycling (85.0 (62.2, 93.8)) also demonstrated significantly better CERT reporting than NMES studies (50.00 (39.5, 70.3), *p* = 0.012), progressive mobility studies (54.1 (47.4, 65.0), *p* = 0.010), and multicomponent studies (50.0% (41.8, 59.2), *p* = 0.002). Passive/active intervention studies had significantly better reporting (66.9% (56.7, 77.5)) than multicomponent studies (50.0% (41.8, 59.2), *p* = 0.009). Studies assessed with STROBE achieved significantly higher CERT reporting scores than those assessed with CONSORT (68.6% vs. 52.0%, *p* = 0.014). Additional file 2: Table S5 details CERT reporting for each study.

#### Intervention and control group reporting

We assessed intervention and control groups in 57 (65.5%) studies. Overall, PR intervention group reporting was higher than control groups (median 68.4% (55.6, 80.0) vs. 38.9% (21.1, 55.0), *p* < 0.001)). NMES, progressive mobility, and multicomponent studies all reported their intervention groups significantly better than their controls with median (*p* value) reporting scores of 65.5% vs. 26.9% (*p* < 0.001), 73.7% vs. 30.0% (*p* < 0.001), and 63.2% vs. 36.8% (*p* < 0.001), respectively.

## Discussion

The first ICUs were established in the late 1950s [75] with a distinct focus on survival. With advancing technology and improving survivorship, there is a shift toward evaluating interventions to improve morbidity. The first prospective original study evaluating an ICU PR intervention was published in 1984, 34 years after the first ICUs. The majority of ICU PR research emerged after 2010, with 2.5 times the number of studies and 3 times the number of RCTs by the end of 2016 (Fig. 2a). Our scoping review assessed the types and amounts of PR received by patients in ICU and evaluated the completeness of reporting in 117 studies. No study evaluated the same intervention in the same way. Thirty-seven percent of studies did not report intervention start time and 26% did not report overall duration. Overall study reporting was adequate; however, PR intervention reporting was substandard. These reporting deficiencies limit our understanding of current ICU PR interventions. For the field of ICU PR to advance, intervention reporting must improve.

### Overall study reporting

We identified no significant differences in study reporting across the guidelines. Transparent reporting of randomized study designs is important because they are the most methodologically rigorous to inform clinical treatment decisions, and their results can inform the design of new studies. Studies assessed with CONSORT demonstrated adequate reporting with a median score of 75%; however, there was a wide range of scores from 31 to 100%. These data identify opportunities for improvement, particularly in interventions, harms, and sample size calculations. Inadequate reporting impairs risk of bias assessments; improved study reporting will allow better assessment of risk of bias and improve our confidence in interpreting study results. Therefore, improving reporting of these RCTs is an important step to advance the field.

### Intervention reporting

Studies could achieve good study reporting scores but still have poorly reported interventions, which impairs the utility of published studies. The quality of intervention reporting assessed by CERT was highly variable with scores from 0 [38] to 100% [76]. Both CONSORT and SQUIRE represent the intervention as 1 item; however, detailed information about the intended interventions, dose, and treatment fidelity are required for replication, especially in complex PR interventions. CERT was developed to address this gap, provides more explicit direction, and facilitates more granular evaluation of intervention reporting [19]. A recent systematic review evaluated 16 RCTs of early PR in the ICU [77] and is, to our knowledge, the only study to use CERT in this population. Authors concluded that intervention reporting was inadequate with a mean CERT reporting score of 61% [77], which was similar to the 56% median score in this study. Among the studies included in our review, the most common intervention reporting limitations included adherence measurement, motivation strategies, decision rules (starting, exercise progression), how exercises were progressed, intensity, tailoring, and fidelity.

Intervention fidelity is a critical aspect of trial reporting. Fidelity describes the extent to which an intervention was delivered as planned [78]. In our study, less than half of all studies reported fidelity (CERT item 16b). Fidelity helps distinguish between implementation failure and intervention failure [78]. It can also provide information on intervention tailoring to fit a particular context (or patient) compared to changes that may undermine fidelity such as substantial deviations from the protocol [78]. For example, a recent RCT of intensive vs. standard rehabilitation reported no difference in the physical component summary measure of the SF-36 at 6 months [9]. The investigators intended to provide 90 min of PR per day to the intervention group, but only delivered a median of 23 min, representing implementation failure [9]. Fidelity is not discretely assessed in CONSORT or SQUIRE, though items including “implementation of the intervention” (from CONSORT) [79], “study of the intervention,” and “results” (items 9a, b and 13a, b, respectively from SQUIRE) [80] address it indirectly. In contrast, CERT expressly addresses fidelity in 2 items—how fidelity was assessed (item 16a), and how well the intervention was delivered as planned (item 16b).

### Intervention and control group reporting

To our knowledge, we are the first to evaluate intervention and control groups using CERT for critical care PR studies. Detail about the intervention and control groups allows the reader to assess the success of protocol implementation and inform future research [16, 18]. Across the 57 two-group studies in our review, intervention groups were significantly better reported than controls. However, key information was still missing in both groups. In the intervention groups, 4 items, adherence measurement, motivation strategies, progression decision rules, and fidelity measurement were poorly reported.

Substantially more information was missing for control groups, with 11 of the 22 items poorly reported. Only 43% of studies provided enough information to replicate control groups. No study reported the planned control group parameters well. Only half of the studies reported intervention timing and duration, 62% reported frequency, 38% reported intensity, and 37% reported fidelity. Our findings are similar to a systematic review of 200 physiotherapy RCTs [81], where only 25% of the control groups described more than half of the items for TIDier (Template for Intervention Description and Replication) [81]. Similar to our findings, the poorest reported items included dose, intensity, planned tailoring, and fidelity [81]. Missing information on control groups impairs our ability to understand the separation between groups, which is important as PR in the ICU becomes more common.

Progressive mobility and multicomponent interventions, the two most common study types, had the weakest CERT reporting, at 54% and 50%, respectively. One potential reason for this disparity may be the complexity of these interventions. Several key factors characterize complex interventions including the number of interacting components within the intervention and control groups, the number and variability in the outcomes, and the degree of tailoring of the intervention permitted [82]. Tailoring adjusts interventions according to several factors including patient abilities, preferences, comorbidities, and any restrictions [19], and poses the most difficult challenge for ICU PR research reporting. Adjusting for patient ability is an important consideration for patients with critical illness because those with similar baseline characteristics have different recovery trajectories and therefore different abilities at different times [83]. For example, in a multicomponent RCT of intensive physical therapy vs. standard of care physical therapy [8], more than 25% of patients were not able to perform standing exercises during their treatment period [8]. Patients in this study likely required variable amounts of intervention tailoring to suit their needs, ability levels, and goals. In contrast, single interventions, such as passive/active exercises or cycling, would likely require only basic tailoring (e.g., increasing or decreasing the resistance on the cycle ergometer) and can be more easily described and defined.

### Implications of the current state of the science in ICU PR

The gaps in the existing ICU PR literature have important implications for clinical practice and future research. Most studies were from the USA, were single-centered, and enrolled small sample sizes, leading to limited generalizability to other countries, centers, and patients. The average age of enrolled patients was 60 years old; with an aging population, we also need to evaluate interventions with older adults.

We believe the most important gap was poor intervention and control group reporting. Intervention-specific reporting guidelines are fairly recent. CERT was first published in 2016, [19] and authors or journal editors may not be aware of these new reporting guidelines yet. CONSORT, first published in 1996, is now endorsed by more than 600 journals. Even 17 years later, a Cochrane review demonstrated that overall RCT reporting was still sub-optimal [84]. As ICU PR becomes more common, complete intervention and control group reporting is critical. For clinicians, inadequate information on the frequency, intensity, type, and time of PR limits their ability to implement published interventions. For researchers, poor reporting leads to challenges reproducing a study’s protocol and limits the foundation to design new trials. Missing information on control groups also limits our interpretation of study results, as we are not able to discern separation between groups. Conversely, accurate, complete, and transparent reporting facilitates replication and is necessary to minimize waste in the time and resource investment in research [85].

We suggest several ways to improve study reporting including the use of intervention-specific reporting guidelines such as CERT. In study planning phases, CERT could inform the development of data collection forms; this would assist with minimizing missing data that may be challenging to obtain post hoc (e.g., information on tailoring). Granting agencies could consider the use of CERT for standardized review of complex rehabilitation interventions. Authors can use CERT in conjunction with the corresponding reporting guidelines for manuscript preparation. Finally, CERT could guide journal editors and reviewers for manuscript submission and review criteria. High quality reporting to inform future research is critical; particular attention is needed for intervention and control groups.

### Limitations and strengths

Our review has limitations. Given the large number of citations retrieved and the number of included studies, we excluded non-English language studies for feasibility. As a result, our report may not reflect PR interventions reported in other languages. We excluded grey literature such as conference abstracts; however, these documents have stringent word count limitations and would not likely report information necessary for accurate study evaluation [86]. Furthermore, there is a lack of central sources for grey literature leading to challenges locating potentially relevant citations [86]. To evaluate the completeness of reporting, we assigned all items in each reporting guideline the same weight. Some items may be considered more relevant than others, which may influence the interpretation of the scores.

Our study also has several important strengths. Our scoping review broadly examines the state of the literature, in contrast to systematic reviews that address a narrow research question. We evaluated a breadth of the literature, including quality improvement and observational studies, which often form the basis to develop larger randomized trials. We developed a comprehensive search strategy and searched five electronic databases from inception to December 2016. We used a rigorous methodology to optimize reviewer agreement and data quality. We complemented overall study reporting by CONSORT, STROBE, or SQUIRE with granular assessment of ICU PR interventions using CERT. In a novel use of CERT, we evaluated intervention and control groups separately. Finally, we identified important opportunities to improve ICU PR intervention and control group reporting.

## Conclusions

PR in ICU is a burgeoning field of research. Our review sought to synthesize and evaluate the nature and extent of prospective original research in the area. We identified a heterogeneous body of literature evaluating a variety of different interventions, ICU settings, and patient populations. Our most important finding was the critical gaps in ICU PR intervention reporting, which limits our understanding of current ICU PR interventions. Given the widespread interest in improving patients’ outcomes, ICU PR is a promising intervention; however, intervention and control group reporting needs to improve for our field to advance. With the utility of published studies resting on a foundation of complete reporting, researchers can use results from this study to inform reporting and conduct of future ICU PR studies.

## Additional files

Additional file 1: Electronic Supplement for Physical Rehabilitation in the ICU Scoping Review. This online data supplement includes the following: Table S1. Electronic search strategy. Table S2. Descriptions of intervention categories. Table S3. Decision rules for scoring using reporting guidelines. Table S4. Overview of interventions reported in 117 studies. Figure S1a. Overview of 20 single component inter-patient RCTs with intervention start times and duration. Figure S1b. Overview of 12 multicomponent inter-patient RCTs with intervention start times and duration. (DOCX 412 kb)

Additional file 2: Table S5. Consensus on Exercise Reporting Template reporting scores for eligible studies (*n* = 87). This supplemental table includes the intervention reporting scores for 87 studies using the Consensus on Exercise Reporting Template scoring. (XLSX 560 kb)

## Abbreviations

CERT: Consensus on Exercise Reporting Template
CONSORT: Consolidated Standards of Reporting Trials
ICU: Intensive care unit
MV: Mechanical ventilation
NMES: Neuromuscular electrical stimulation
PR: Physical rehabilitation
RCT: Randomized clinical trial
SQUIRE: Standards for Quality Improvement Reporting Excellence
STROBE: Strengthening the Reporting of Observational Studies in Epidemiology
