# Supplementary material for: Physical rehabilitation interventions in the intensive care unit: a scoping review of 117 studies
Source: J Intensive Care. 2018 Dec 7;6:80. doi: 10.1186/s40560-018-0349-x (PMC6286501; doi:10.1186/s40560-018-0349-x)
Supplement: Supplementary file 1 — Electronic Supplement for Physical Rehabilitation in the ICU Scoping Review. This online data supplement includes the following: Table S1. Electronic search strategy. Table S2. Descriptions of intervention categories. Table S3. Decision rules for scoring using reporting guidelines. Table S4. Overview of interventions reported in 117 studies. Figure S1a. Overview of 20 single component inter-patient RCTs with intervention start times and duration. Figure S1b. Overview of 12 multicomponent inter-patient RCTs with intervention start times and duration. (DOCX 412 kb) [file 40560_2018_349_MOESM1_ESM.docx]

**Title:** Physical Rehabilitation Interventions in the Intensive Care Unit: A Scoping Review of 117 Studies

**Authors:**

Julie C Reid MSc PT, PhD candidate

Janelle Unger MSc PT, PhD candidate

Devin McCaskell BSc

Laura Childerhose BSc (Hons)

David J Zorko MD

Michelle E Kho PT, PhD

**Online Data Supplement**

**Table S1:** Electronic search strategy developed by authors in consultation with health research librarian.

1. OVID Medline

|  | **Subject Headings (MeSH)** | **Key Words** |
| --- | --- | --- |
| 1. ICU subject headings and keywords | Intensive care units  Critical illness  Critical care  Respiration, artificial  Respiratory care units | ICU  ICU patient*  Acute care  Critical care  Critical care unit*  Mechanical ventilation  Intensive care unit*  Intensive care  Respiratory care unit*  Critical* ill*  Intubated  Ventilated patient* |
| 2. Physical activity subject headings and keywords | Mobility limitation  Rehabilitation  Rehabilitation, vocational  Physical and rehabilitation medicine  Exercise therapy  Exercise  Physical therapy modalities  Range of motion, articular  Activities of daily living  Recovery of function  Muscle strength | Mobili*  Early mobili*  Functional mobili*  Physical therap*  Physiotherap*  Physical function*  Exercis*  Exercise therap*  Activit* of daily living  ADL  Rehab*  Rehab* intervention*  Range of motion  ROM  Recovery of function  Muscle strength  Physical and rehab* medicine  Physical activit*  Physical mobili*  Early ambulation  Recovery of function |

1. We combined all ICU subject headings and keywords with the OR operator

2. We combined all physical rehabilitation subject headings and keywords with the OR operator

3. We combined these 2 groups with the AND operator

2) Cumulative Index to Nursing and Allied Health Literature (CINAHL)

|  | **Subject Headings** | **Keywords** |
| --- | --- | --- |
| 1. ICU subject headings and key words | Intensive Care Units  Critical care  Respiratory care Units  Respiration, artificial  Critical illness  Critically ill patients | ICU  ICU patient*  Intensive care unit*  Intensive care  Critical care unit*  Acute care  Critical care  Critical* ill*  Intubated  Ventilated patient*  Mechanical ventilation  Respiratory care unit* |
| 2. Physical rehabilitation subject headings and keywords | Exercise  Therapeutic exercise  Physical activity  Physical therapy  Rehabilitation  Muscle strength  Activities of daily living  Early intervention | Physical activit*  Exercise therap*  Exercis*  Physiotherap*  Physical therap*  Recovery of function  Physical function*  Rehab*  Physical mobili*  Early ambulation  Early mobili*  Range of motion  Functional mobili*  Rehab* intervention*  Muscle strength  Activit* of daily living  ADL  Mobili*  ROM  Recovery of function  Muscle strength  Physical and rehab* medicine |

1. We combined all ICU subject headings and keywords with the OR operator

2. We combined all physical rehabilitation subject headings and keywords with the OR operator

3. We combined these 2 groups with the AND operator

3) Allied and Complimentary Medicine Database (AMED)

|  | **Subject Heading** | **Keywords** |
| --- | --- | --- |
| 1. ICU subject headings and keywords | Intensive care  Critical care  Critical illness  Respiration artificial  Ventilators, mechanical | ICU  Intensive care unit*  Critical* ill*  Acute care  Mechanical ventilation  Respiratory care unit*  ICU patient*  Intensive care  Critical care unit*  Critical care  Intubated  Ventilated patient* |
| 2. Physical rehabilitation subject headings and keywords | Rehabilitation  Physical therapy modalities  Muscle strength  Range of motion  Exercise  Exercise therapy  Physiotherapists  Mobility limitation  Early ambulation  Activities of daily living | Exercis*  Physiotherap*  Physical therap*  Early mobili*  Functional mobili*  Rehab*  Rehab* intervention*  Physical activit*  Exercise therap*  Recovery of function  Physical function*  Physical mobili*  Early ambulation  Range of motion  Muscle strength  Activit* of daily living  ADL  Mobili*  ROM  Recovery of function  Muscle strength  Physical and rehab* medicine |

1. We combined all ICU subject headings and keywords with the OR operator

2. We combined all physical rehabilitation subject headings and keywords with the OR operator

3. We combined these 2 groups with the AND operator

4) Excerpta Medica Database (EMBASE)

|  | **Subject Headings** | **Keywords** |
| --- | --- | --- |
| 1. ICU subject headings and keywords | Intensive care unit  Intensive care  Critical illness  Artificial ventilation | ICU  Intensive care unit*  Intensive care  Critical* ill*  Mechanical ventilation  Respiratory care unit*  ICU patient*  Critical care unit*  Acute care  Critical care  Intubated  Ventilated patient* |
| 2. Physical rehabilitation subject headings and keywords | Muscle strength  Rehabilitation  Vocational rehabilitation  Physical activity  Exercise  Kinesiotherapy  Physiotherapy  Mobilization  Daily life activity | Physical activit*  Exercis*  Exercise therap*  Physical therap*  Recovery of function  Physical mobili*  Physical function*  Early ambulation  Early mobili*  Functional mobili*  Range of motion  Rehab*  Rehab* intervention*  Activit* of daily living  Physiotherap*  Muscle strength  ADL  Mobili*  ROM  Physical and rehab* medicine |

1. We combined all ICU subject headings and keywords with the OR operator

2. We combined all physical rehabilitation subject headings and keywords with the OR operator

3. We combined these 2 groups with the AND operator

5) Physiotherapy Evidence Database (PEDro)

| (intensive care unit OR critical care OR critical illness) |
| --- |
| (intensive care unit* AND electrotherapies [“Therapy “ field]) |
| (critical ill* AND “stretching, mobilisation, etc.” [Therapy field]) |
| (intensive care unit* AND “stretching, mobilisation, etc.” [Therapy field]) |

**Table S2:** Criteria and intervention descriptions for each category

| **Intervention Category** | **Definition/Criteria** |
| --- | --- |
| Neuromuscular electrical stimulation (NMES) | Application of electrical stimulation via electrodes attached to the skin over pre-determined muscles to elicit a muscle contraction. May include passive only (i.e., no active participation from the patient) or active muscle contractions simulatenously |
| Passive/active exercises | Includes activites such as range of motion (passive or active), positioning in bed, sitting only activities, etc. but that are not part of a progressive mobility regimen |
| Cycling | May include upper or lower extremity cycle erogmetry, either passively completed by the equipment, components of active participation from the patient, or full participation from the patient |
| Progressive mobility | Mobility activities progressing from less difficult (e.g., activity in bed) to more difficult (e.g., out of bed activities such as up to chair or ambulation). May include range of motion or strengthening activities, but only as part of a mobility activity and not as a separate therapeutic program |
| Multi-component | Includes at least 2 or more interventions which could include any combination of single component interventions above and other intervention such as respiratory-related, etc. |

**Table S3:** Decision rules for applying “not applicable” when evaluating CONSORT, STROBE, SQUIRE, and CERT reporting items.

| **Item Number** | **Item Description** | **Examples where this item was not applicable** |
| --- | --- | --- |
| CONSORT |  |  |
| 3b | Changes to methods or trial design after trial commenced | Studies that did not make changes to methods |
| 6b | Any changes to trial outcomes after the trial commenced, with reasons | Studies that evaluated the planned outcomes as planned in the methods |
| 7b | Interim analyses | Studies that did not conduct interim analyses |
| 12b | Additional analyses | Studies that did not plan to perform subgroup or adjusted analyses |
| 17b | Outcomes and estimation – presentation of absolute and relative risk size for binary outcomes | Studies that did not evaluate binary outcomes |
| 18 | Ancillary analyses | Studies that did not perform subgroup or adjusted analyses |
| STROBE | | |
| 12b | Describe statistical methods used to examine subgroups | Studies that did not evaluate/analyze subgroups |
| 12d | Describe how loss to follow up was addressed | Studies that did not report any loss to follow up |
| 14c | Summarize follow up time | Studies that did not have a follow up period |
| CERT | | |
| 3 | Describe whether exercises performed individually or in a group | We assumed that all interventions in the ICU were performed individually |
| 5 | Describe how adherence to exercise is measured and reported | Studies evaluating an intervention consisting of a single session |
| 6 | Describe motivation strategies | Studies evaluating passive interventions (e.g., NMES) |
| 7a | Describe decision rules for determining exercise progression | Studies evaluating passive interventions (e.g., passive ROM) |
| 7b | Describe how the exercise program was progressed | Studies evaluating passive interventions (e.g., passive ROM) |
| 9 | Describe any home program content | Studies that did not implement a home program |
| 10 | Describe whether there are any non-exercise components | Studies that did not implement a non-exercise component |
| 14b | Describe how exercises are tailored to the individual | Studies that did not individually tailor interventions |

Legend: CONSORT – Consolidated Standards of Reporting Trials; STROBE – Strengthening the Reporting of Observational Studies in Epidemiology; SQUIRE – Standards for Quality Improvement Reporting Excellence; CERT – Consensus on Exercise Reporting Template; NMES – neuromuscular electrical stimulation; ROM – range of motion. Table E3: Decision rules for applying not applicable to assessment of reporting guideline adherence. We determined denominators by subtracting all not applicable items from total items.

**Table S4:** Overview of interventions reported in 117 studies of ICU physical rehabilitation interventions. Citations presented herein are listed at the end of the online supplement.

|  |  |  |  |
| --- | --- | --- | --- |

| **Single Interventions** | **Enrolled (n)** | **Intervention Group Description** | **Frequency, Intensity, Time** | **Comparison Group Description** | **Frequency, Intensity, Time** |
| --- | --- | --- | --- | --- | --- |
| **NMES** |  |  |  |  |  |
| RCTs (n=14)  [1-14] | 541 | Stimulated muscles: unilateral or bilateral; quadriceps (n=13), lower leg muscles (tibialis anterior, gastrocnemius, peroneus longus, n=4), gluteus muscles (n=1), biceps brachii (n=1), accessory respiratory muscles (pectoralis major, trapezius, latissimus dorsi, n=1)  Settings: 50-100 Hz, 0-150mA, 200-400 μsec pulse duration  Co-interventions, when applicable were applied to both groups and included “PT”, passive ROM, and mobilization (head of bed up positioning, sitting edge of bed) | -Frequency range from 1-2x daily  -Duration range from 20-60 minute sessions  -Intensities range from visible contraction to palpable contraction and pain tolerance | NMES comparators included moderate frequency NMES, sham control (including stimulation until tingling felt but no contraction), no treatment provided to comparator group, and not reported | -Frequency range from not reported, single session, to 1-3 x daily, and 10 repetitions per movement (for ROM)  -Duration range from not reported to 10-60 minutes |
| 2-group comparison (n=2)  [15 16] | 52 | Stimulated muscles: bilateral quadriceps (n=2), lower leg muscles (biceps and triceps surae, tibialis anterior, peroneus longus, n=1)  Settings range: 45 Hz, 30-80mA, 400 μsec pulse duration | -Frequency range from single session to daily sessions  -30 – 55 minutes per session  -Intensity: to visible contraction | Included no treatment to passive ROM | Not reported |
| Case Series (n=1)  [17] | 50 | Stimulated muscles: bilateral quadriceps femoris  Settings: 50 Hz, 0-80mA, 300-500 μsec pulse duration | -5 days/week, 25 minutes/day, until ICU discharge  -Intensity: to palpable and visible contraction | N/A | N/A |
| **Passive or active exercises** |  |  |  |  |  |
| RCTs (n=5)  [18-22] | 170 | Exercises included bilateral upper extremity passive ROM, passive slide transfer to seated position out of bed, and tilt table verticalization with passive robotic stepping  Included assisted transfer supine to sitting at the edge of the bed to assisted transfer from bed to armchair | -Frequency range from single session to 2x/day, 5 days/week  -Duration range from 15-minutes to 30-minutes  -Sitting time ranged from 10-minutes to 120 minutes | Included bilateral upper extremity passive ROM started 2 weeks after the intervention group, passive positioning in the semi-recumbent position in bed, and mobilization exercises supine or sitting on bed  Included lying comfortably in bed (position between supine and semi-recumbent) to slide transferring to a lateral transfer chair then being positioned in upright | -Frequency range from not reported to single session to 2x/day, 5 days/week  -Duration range from 10-minutes to 120-minutes |
| 2-group comparison (n=2)  [23 24] | 83 | Unilateral lower extremity passive ROM and patient positioning with Patient Positioning System | -Single session  -7 repetitions/ movement  -2 hours positioned on side | Unilateral lower extremity passive ROM in healthy controls and standard of care positioning | -Single session  -7 repetitions/ movement  -2 hours positioned on side |
| Before-After (n=1)  [25] | 32 | Bilateral lower extremity passive ROM | -Single session  -20 minutes duration | Bed rest (pre-ROM exercises) | -Single session  -30-minutes |
| Case Series (n=8)  [26-33] | 235 | Exercises included passive tilt table standing (n=2), passive ROM, passive lateral decubitus positioning, shoulder flexion, and isometric hand squeezing exercises | -Frequency range from not reported to single session to daily sessions  -Duration range from not reported to 4 minutes – 30 minutes duration and/or 10 repetitions/ movement | N/A | N/A |
| Cohort (n=2)  [34 35] | 100 | Included passive transfer to a chair, sitting edge of the bed with active patient participation, and positioning and/or suctioning procedures | Frequency range from not reported to single session | N/A | N/A |
| **Cycling** |  |  |  |  |  |
| RCT (n=3)*  [36-38] | 145 | Passive or active in-bed leg cycling in addition to individualized respiratory physiotherapy, standardized mobilization of upper and lower extremities, and PNF techniques | -Frequency range from not reported to 5 days/week  -20-30 minutes cycling/day (could be split into 2 sessions)  -Intensity: resistance level increased each sessions as tolerated | Individualized respiratory physiotherapy, standardized mobilization of upper and lower extremities, PNF techniques, and daily in-bed mobility | -Frequency range from not reported to 5 days/week  -Duration range from not reported to 30 minutes  -Intensity increased according to patients’ capability |
| Case Series (n=5)  [39-43] | 171 | Included passive leg cycling at 20-30 revolutions per minute, 0 watts, to active cycling at stable pace against 3 watts or 6 watts resistance to cycling as fast as possible (maintaining same pace x 5 minutes) | -Frequency range from single session to 6 days/week  -Duration from 5-30 minutes | N/A | N/A |
| **Progressive mobility** |  |  |  |  |  |
| RCTs (n=5)  [44-48] | 487 | Activities included head of bed up, supine to sit transfers, sitting edge of bed, sit to stand transfers, ambulation  -Use of specialized mobility equipment to improve efficiency, effectiveness, and safety | -Frequency range from not reported - 2x daily  -Time and intensity ranged from not reported to “per patient condition” to 60 minutes | Activities included passive movements, assisted progressive mobility post-ICU discharge, “routine treatment”, and ambulation using standard mobility equipment | -Frequency range from not reported to daily  -Duration range from not reported to 5-10 minutes |
| 2-group comparison (n=1)  [49] | 330 | Activities included passive ROM to active-assisted and active ROM, sitting position in bed, sitting edge of bed, active transfer to chair, ambulation | -Daily, 7days/week  -5 repetitions/joint for ROM, 3x/day for sitting position, 1x/day for out of bed to chair  -Sitting activities x 20 minutes  -Intensity dependent on patient level of consciousness | Included infrequent and irregular delivery of PT interventions to RN-led passive ROM and positioning | -Daily passive ROM and positioning  -Every 2-hours positioning |
| Cohort (n=11)  [50-60] | 2,119 | Activities included in-bed positioning, passive/active ROM, passive and active limb exercises, sitting, standing, and ambulation | -Frequency range from not reported to every 2 hours to 2x daily  -Duration range from not reported – 30-60 minute sessions  -Intensity not reported | RN-led mobility activities including bed mobility, sitting edge of bed, out of bed to chair, and gait training | Not reported |
| Before-After (n=12)  [61-72] | 3,977 | Activities included positioning in bed, passive and active limb exercises, passive transfer to chair, sitting edge of bed, standing, marching on the spot, and ambulation  Implementation activities included staff education, mobility champions, mobility sub-teams, custom equipment (e.g., custom walkers), mobility decision algorithms, progression protocols, safety guidelines, guidelines for consulting PT/OT, mandatory mobility order sets, and increasing rehabilitation staffing | -Frequency range from 1-3x daily as patient tolerates  -Duration range from 20-60 minutes | Included no dedicated PT service unless ordered by a physician, bed rest as standard mobility order, RN-led positioning and ROM, and usual care (some of which included progressive mobility activities) | -Frequency range from not reported to daily  -Duration range from not reported to 20-30 minutes |
| Case Series (n=9)  [73-81] | 463 | Activities included passive ROM, passive and active limb exercises, sitting balance exercises, chair sitting, standing, tilt table standing (n=1), marching on the spot, and ambulation (including 6-minute walk test) | -Frequency range from not reported to single session to 2x daily  -Duration range from 10 minutes – 2 hour sessions  -Intensity range from not reported to “based on PFIT-s results from previous day” | N/A | N/A |
| **Multicomponent Interventions** |  |  |  |  |  |
| RCTs (n=12)  [82-93] | 1,220 | Interventions included:  -progressive mobility (n=10)  -NMES (n=1)  -passive/active exercises (n=5)  -leg cycling (n=3)  Other interventions included: respiratory interventions** (n=5), strengthening programs (including respiratory muscle strengthening, n=7), balance training, cognitive interventions, and ADL training | -Frequency range from not reported up to 3xdaily  -Duration range from not reported to 15-45 minutes  -Intensity: range from not reported to 60-80% age-predicted heart rate maximum or Borg scale 3-5 | Interventions included no rehabilitation per any protocol but could be ordered by ICU physicians, passive/active exercises, progressive mobility  -could also include same intervention as the experimental group but administered at a different frequency | -Frequency range from not reported, 1-3 sessions/week, up to daily  -Duration range from 0-23 minutes |
| 2-group comparison (n=4)  [94-97] | 845 | Interventions included:  -NMES (n=1)  -passive/active limb exercise (n=1)  -leg cycling (n=1)  -progressive mobility (n=3)  Other interventions included: respiratory interventions** (n=3) | -Patients assessed daily  -Frequency range from not reported to 1-2x daily  -Duration range from not reported to 20-60 minutes  -Intensity not reported | Interventions included: based on clinical decision of PT responsible for care, standard nursing care, passive joint exercises, progressive mobility (sitting edge of bed through to ambulation) | -Patients assessed daily  -Intervention frequency based on clinical decision of PT responsible for care  -Duration range from not reported to 5-15 minutes |
| Cohort (n=11)  [98-108] | 2,402 | Interventions included:  -NMES (n=1)  -passive/active limb exercises (n=8)  -leg cycling (n=3)  -progressive mobility (n=9)  Other interventions included: respiratory interventions** (n=5), ADL training (n=1), video game interventions (n=1), multisensory stimulation (n=1), education (n=1), global kinesiotherapy (n=1) | -Frequency range from not reported to 2x/day  -Duration range from not reported to 15-40 minute sessions | Included no comparator intervention to standard individualized physiotherapy | Not reported |
| Before-after (n=1)  [109] |  | Interventions included passive/active limb exercises and progressive mobility  Other interventions included arm and leg cycling | -Frequency range from every 2 hours for positioning to 3x/day for mobility  -Up to 30 minutes/session | Not reported | Not reported |
| Case Series (n=7)  [110-116] | 400 | Interventions included:  -passive/active limb exercises (n=7)  -arm and leg cycling (n=2)  -progressive mobility (n=5)  Other interventions included: education (n=2), strengthening programs (including respiratory muscle training, n=3), respiratory interventions** (n=3), PNF patterns (n=1), tilt table standing (n=1) | -Frequency range from not reported to daily screening up to 2x/day intervention delivery  -Duration range from not reported up to 30 minutes | N/A | N/A |
| **Unclassified intervention** |  |  |  |  |  |
| Cohort (n=1)  [117] | 386 | Interventions included:  Not reported | -Frequency, duration, intensity not reported | N/A | N/A |

Legend: RCT – randomized clinical trial; NMES – neuromuscular electrical stimulation; PT – physiotherapy; ROM – range of motion; ICU – intensive care unit; RN – registered nurse; OT – occupational therapy; PFIT-s – Physical Function Test for ICU-scored; ADL –activities of daily living; PNF – proprioceptive neuromuscular facilitation. *1 cycling RCT had 2 intervention arms: leg cycling and passive verticalization with passive stepping. **Respiratory interventions included, but were not limited to, bronchial drainage and suctioning, breathing exercises, manual lung hyperinflation, coughing, thoracic expansion exercises, chest physiotherapy (percussions/vibrations).


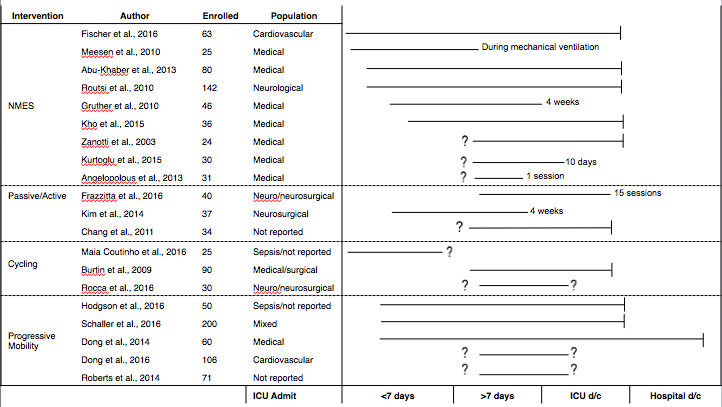


**Figure S1a** In this figure we present 20 single component inter-patient RCTs by intervention category showing intervention start times and duration

Legend: RCT – randomized clinical trial; ? – start time or duration not reported; arrows indicate that the intervention extends beyond the hospital stay.


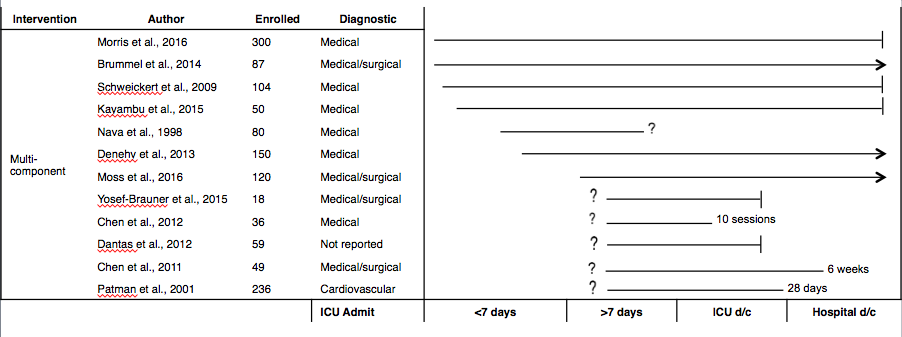


**Figure S1b** In this figure we present 12 multicomponent inter-patient RCTs showing intervention start times and duration.

Legend: RCT – randomized clinical trial; ? – start time or duration not reported; arrows indicate that the intervention extends beyond the hospital stay.

**Included Study References:**

1. Fischer A, Spiegl M, Altmann K, et al. Muscle mass, strength and functional outcomes in critically ill patients after cardiothoracic surgery: does neuromuscular electrical stimulation help? The Catastim 2 randomized controlled trial. Critical Care (London, England) 2016;**20**(1):30

2. Kho ME, Truong AD, Zanni JM, et al. Neuromuscular electrical stimulation in mechanically ventilated patients: a randomized, sham-controlled pilot trial with blinded outcome assessment. Journal of Critical Care 2015;**30**(1):32-9

3. Kocan Kurtoglu D, Tastekin N, Birtane M, Tabakoglu E, Sut N. Effectiveness of neuromuscular electrical stimulation on auxiliary respiratory muscles in patients with chronic obstructive pulmonary disease treated in the intensive care unit. Turkiye Fiziksel Tip ve Rehabilitasyon Dergisi 2015;**61**(1):12-17

4. Falavigna LF, Silva MG, Freitas AL, et al. Effects of electrical muscle stimulation early in the quadriceps and tibialis anterior muscle of critically ill patients. Physiotherapy theory and practice 2014;**30**(4):223-28

5. Abu-Khaber HA, Abouelela AMZ, Abdelkarim EM. Effect of electrical muscle stimulation on prevention of ICU acquired muscle weakness and facilitating weaning from mechanical ventilation. Alexandria Journal of Medicine 2013;**49**(4):309-15

6. Angelopoulos E, Karatzanos E, Dimopoulos S, et al. Acute microcirculatory effects of medium frequency versus high frequency neuromuscular electrical stimulation in critically ill patients - a pilot study. Annals of Intensive Care 2013;**3**(1):1-9

7. Poulsen JB, Moller K, Jensen CV, Weisdorf S, Kehlet H, Perner A. Effect of transcutaneous electrical muscle stimulation on muscle volume in patients with septic shock. Critical Care Medicine 2011;**39**(3):456-61

8. Gruther W, Kainberger F, Fialka-Moser V, et al. Effects of neuromuscular electrical stimulation on muscle layer thickness of knee extensor muscles in intensive care unit patients: a pilot study. Journal of Rehabilitation Medicine 2010;**42**(6):593-7

9. Meesen RL, Dendale P, Cuypers K, et al. Neuromuscular electrical stimulation as a possible means to prevent muscle tissue wasting in artificially ventilated and sedated patients in the intensive care unit: A pilot study. Neuromodulation 2010;**13**(4):315-20; discussion 21 doi: 10.1111/j.1525-1403.2010.00294.x[published Online First: Epub Date]|.

10. Routsi C, Gerovasili V, Vasileiadis I, et al. Electrical muscle stimulation prevents critical illness polyneuromyopathy: a randomized parallel intervention trial. Critical Care (London, England) 2010;**14**(2):R74

11. Zanotti E, Felicetti G, Maini M, Fracchia C. Peripheral muscle strength training in bed-bound patients with COPD receiving mechanical ventilation: effect of electrical stimulation. Chest 2003;**124**(1):292-6

12. Bouletreau P, Patricot MC, Saudin F, Guiraud M, Mathian B. Effects of intermittent electrical stimulations on muscle catabolism in intensive care patients. Jpen: Journal of Parenteral & Enteral Nutrition 1987;**11**(6):552-5

13. Dirks ML, Hansen D, Van Assche A, Dendale P, Van Loon LJ. Neuromuscular electrical stimulation prevents muscle wasting in critically ill comatose patients. Clinical Science 2015;**128**(6):357-65

14. Rodriguez PO, Setten M, Maskin LP, et al. Muscle weakness in septic patients requiring mechanical ventilation: protective effect of transcutaneous neuromuscular electrical stimulation. Journal of Critical Care 2012;**27**(3):319.e1-8

15. Gerovasili V, Tripodaki E, Karatzanos E, et al. Short-term systemic effect of electrical muscle stimulation in critically ill patients. Chest 2009;**136**(5):1249-56

16. Hirose T, Shiozaki T, Shimizu K, et al. The effect of electrical muscle stimulation on the prevention of disuse muscle atrophy in patients with consciousness disturbance in the intensive care unit. Journal of Critical Care 2013;**28**(4):536.e1-7

17. Segers J, Hermans G, Bruyninckx F, Meyfroidt G, Langer D, Gosselink R. Feasibility of neuromuscular electrical stimulation in critically ill patients. Journal of Critical Care 2014;**29**(6):1082-8

18. Frazzitta G, Zivi I, Valsecchi R, et al. Effectiveness of a very early stepping verticalization protocol in severe acquired brain injured patients: A randomized pilot study in icu. PLoS ONE 2016;**11 (7) (no pagination)**(e0158030)

19. Kim H, Lee Y, Sohng K. Effects of bilateral passive range of motion exercise on the function of upper extremities and activities of daily living in patients with acute stroke. Journal of Physical Therapy Science 2014;**26**(1):149-56.

20. Thomas P, Paratz J, Lipman J. Seated and semi-recumbent positioning of the ventilated intensive care patient - effect on gas exchange, respiratory mechanics and hemodynamics. Heart & Lung 2014;**43**(2):105-11

21. Chang MY, Chang LY, Huang YC, Lin KM, Cheng CH. Chair-sitting exercise intervention does not improve respiratory muscle function in mechanically ventilated intensive care unit patients. Respiratory Care 2011;**56**(10):1533-8

22. Collings N, Cusack R. A repeated measures, randomised cross-over trial, comparing the acute exercise response between passive and active sitting in critically ill patients. BMC Anesthesiology 2015;**15**:1

23. Thelandersson A, Volkmann R, Cider A. Blood flow velocity and vascular resistance during passive leg exercise in the critically ill patient. Clinical Physiology & Functional Imaging 2012;**32**(5):338-42

24. Powers J. Two Methods for Turning and Positioning and the Effect on Pressure Ulcer Development: A Comparison Cohort Study. Journal of wound, ostomy, and continence nursing : official publication of The Wound, Ostomy and Continence Nurses Society 2016;**43**(1):46-50

25. Amidei C, Sole ML. Physiological responses to passive exercise in adults receiving mechanical ventilation. American Journal of Critical Care 2013;**22**(4):337-48

26. Toccolini BF, Osaku EF, de Macedo Costa CRL, et al. Passive orthostatism (tilt table) in critical patients: Clinicophysiologic evaluation. Journal of Critical Care 2015;**30**(3):655.e1-55.e6

27. Roth C, Stitz H, Kalhout A, Kleffmann J, Deinsberger W, Ferbert A. Effect of early physiotherapy on intracranial pressure and cerebral perfusion pressure. Neurocritical Care 2013;**18**(1):33-8

28. Silva RFAd, Nascimento MAdL. Therapeutic mobilization as nursing care: evidence from practice. Revista da Escola de Enfermagem da USP 2012;**46**(2):413-19 7p doi: dx.doi.org/S0080-62342012000200020[published Online First: Epub Date]|.

29. Chang AT, Boots R, Hodges PW, Paratz J. Standing with assistance of a tilt table in intensive care: a survey of Australian physiotherapy practice. Australian Journal of Physiotherapy 2004;**50**(1):51-4

30. Norrenberg M, De Backer D, Freidman G, Moraine J-J, Vincent J-L. Cardiovascular response to passive leg movement in critically ill patients. Clinical Intensive Care 1999;**10**:1-6

31. Brimioulle S, Moraine JJ, Norrenberg D, Kahn RJ. Effects of positioning and exercise on intracranial pressure in a neurosurgical intensive care unit. Physical Therapy 1997;**77**(12):1682-9

32. Koch SM, Fogarty S, Signorino C, Parmley L, Mehlhorn U. Effect of passive range of motion on intracranial pressure in neurosurgical patients. Journal of Critical Care 1996;**11**(4):176-9

33. Richard R, Staley M, Miller SF. The effect of extremity range of motion on vital signs of critically ill patients and patients with burns: a pilot study. Journal of Burn Care & Rehabilitation 1994;**15**(3):281-4

34. Bahadur K, Jones G, Ntoumenopoulos G. An observational study of sitting out of bed in tracheostomised patients in the intensive care unit. Physiotherapy 2008;**94**(4):300-05 6p

35. Robleda G, Roche-Campo F, Membrilla-Martinez L, et al. [Evaluation of pain during mobilization and endotracheal aspiration in critical patients]. Medicina Intensiva 2016;**40**(2):96-104

36. Burtin C, Clerckx B, Robbeets C, et al. Early exercise in critically ill patients enhances short-term functional recovery. Crit Care Med 2009;**37**(9):2499-505 doi: 10.1097/CCM.0b013e3181a38937[published Online First: Epub Date]|.

37. Rocca A, Pignat JM, Berney L, et al. Sympathetic activity and early mobilization in patients in intensive and intermediate care with severe brain injuries: A preliminary prospective randomized study. BMC Neurology 2016;**16 (1) (no pagination)**(169)

38. Maia Coutinho W, Jurema dos Santos L, Fernandes J, Rios Vieira SR, Forgiarini Junior LA, Simões Dias A. Acute effect of the use of cycle ergometer during physical therapy treatment in mechanically ventilated critically ill patients. Fisioterapia e Pesquisa 2016;**23**(3):(6p) doi: 10.1590/1809-2950/15549123032016[published Online First: Epub Date]|.

39. Camargo Pires-Neto R, Fogaca Kawaguchi YM, Sayuri Hirota A, et al. Very Early Passive Cycling Exercise in Mechanically Ventilated Critically Ill Patients: Physiological and Safety Aspects - A Case Series. PLoS ONE 2013;**8 (9) (no pagination)**(e74182)

40. Pires-Neto RC, Pereira AL, Parente C, et al. Characterization of the use of a cycle ergometer to assist in the physical therapy treatment of critically ill patients. Revista Brasileira de Terapia Intensiva 2013;**25**(1):39-43

41. Hickmann CE, Roeseler J, Castanares-Zapatero D, Herrera EI, Mongodin A, Laterre PF. Energy expenditure in the critically ill performing early physical therapy. Intensive Care Medicine 2014;**40**(4):548-55

42. Kho ME, Molloy AJ, Clarke FJ, et al. TryCYCLE: A prospective study of the safety and feasibility of early in-bed cycling in mechanically ventilated patients. PLoS ONE 2016;**11 (12) (no pagination)**(e0167561)

43. Thelandersson A, Nellgard B, Ricksten SE, Cider A. Effects of Early Bedside Cycle Exercise on Intracranial Pressure and Systemic Hemodynamics in Critically Ill Patients in a Neurointensive Care Unit. Neurocritical Care 2016;**25**(3):434-39

44. Dong Z, Yu B, Zhang Q, et al. Early Rehabilitation Therapy Is Beneficial for Patients With Prolonged Mechanical Ventilation After Coronary Artery Bypass Surgery. International Heart Journal 2016;**57**(2):241-6

45. Dong ZH, Yu BX, Sun YB, Fang W, Li L. Effects of early rehabilitation therapy on patients with mechanical ventilation. World journal of emergency medicine 2014;**5**(1):48-52

46. Roberts M, Johnson LA, Lalonde TL. Early mobility in the intensive care unit: Standard equipment vs a mobility platform. American Journal of Critical Care 2014;**23**(6):451-7

47. Schaller SJ, Anstey M, Blobner M, et al. Early, goal-directed mobilisation in the surgical intensive care unit: a randomised controlled trial. The Lancet 2016;**388**(10052):1377-88

48. Hodgson CL, Bailey M, Bellomo R, et al. A binational multicenter pilot feasibility randomized controlled trial of early goal-directed mobilization in the ICU. Critical Care Medicine 2016;**44**(6):1145-52

49. Morris PE, Goad A, Thompson C, et al. Early intensive care unit mobility therapy in the treatment of acute respiratory failure. Crit Care Med 2008;**36**(8):2238-43 doi: 10.1097/CCM.0b013e318180b90e[published Online First: Epub Date]|.

50. Berney SC, Rose JW, Bernhardt J, Denehy L. Prospective observation of physical activity in critically ill patients who were intubated for more than 48 hours. Journal of Critical Care 2015;**30**(4):658-63

51. Harrold ME, Salisbury LG, Webb SA, Allison GT. Early mobilisation in intensive care units in Australia and Scotland: A prospective, observational cohort study examining mobilisation practises and barriers. Critical Care 2015;**19 (1) (no pagination)**(336)

52. Mulkey M, Bena JF, Albert NM. Clinical outcomes of patient mobility in a neuroscience intensive care unit. Journal of Neuroscience Nursing 2014;**46**(3):153-61; quiz E1-2

53. Berney SC, Harrold M, Webb SA, et al. Intensive care unit mobility practices in Australia and New Zealand: a point prevalence study. Critical Care & Resuscitation 2013;**15**(4):260-5

54. Perme C, Nalty T, Winkelman C, Kenji Nawa R, Masud F. Safety and Efficacy of Mobility Interventions in Patients with Femoral Catheters in the ICU: A Prospective Observational Study. Cardiopulmonary Physical Therapy Journal 2013;**24**(2):12-7

55. Hopkins RO, Miller 3rd RR, Rodriguez L, Spuhler V, Thomsen GE. Physical therapy on the wards after early physical activity and mobility in the intensive care unit. Physical therapy 2012;**92**(12):1518-23

56. Leditschke A, Green M, Irvine J, Bissett B, Mitchell IA. What Are the Barriers to Mobilizing Intensive Care Patients? Cardiopulmonary Physical Therapy Journal (American Physical Therapy Association, Cardiopulmonary Section) 2012;**23**(1):26-29 4p

57. Garzon-Serrano J, Ryan C, Waak K, et al. Early mobilization in critically ill patients: patients' mobilization level depends on health care provider's profession. PM & R 2011;**3**(4):307-13

58. Winkelman C, Higgins PA, Chen YJ. Activity in the chronically critically ill. Dimens Crit Care Nurs 2005;**24**(6):281-90

59. Stiller K, Phillips AC, Lambert P. The safety of mobilisation and its effect on haemodynamic and respiratory status of intensive care patients. Physiotherapy Theory & Practice 2004;**20**(3):175-85 11p

60. George AJ, Nair S, Karthic JC, Joseph M. The incidence of deep venous thrombosis in high-risk Indian neurosurgical patients: Need for early chemoprophylaxis? Indian Journal of Critical Care Medicine 2016;**20**(7):412-16

61. Dafoe S, Chapman MJ, Edwards S, Stiller K. Overcoming barriers to the mobilisation of patients in an intensive care unit. Anaesthesia & Intensive Care 2015;**43**(6):719-27

62. Klein K, Mulkey M, Bena JF, Albert NM. Clinical and psychological effects of early mobilization in patients treated in a neurologic ICU: a comparative study. Critical Care Medicine 2015;**43**(4):865-73

63. McWilliams D, Weblin J, Atkins G, et al. Enhancing rehabilitation of mechanically ventilated patients in the intensive care unit: a quality improvement project. Journal of Critical Care 2015;**30**(1):13-8

64. Drolet A, DeJuilio P, Harkless S, et al. Move to improve: the feasibility of using an early mobility protocol to increase ambulation in the intensive and intermediate care settings. Physical Therapy 2013;**93**(2):197-207

65. Mah JW, Staff I, Fichandler D, Butler KL. Resource-efficient mobilization programs in the intensive care unit: who stands to win? American Journal of Surgery 2013;**206**(4):488-93

66. Titsworth WL, Hester J, Correia T, et al. The effect of increased mobility on morbidity in the neurointensive care unit. Journal of Neurosurgery 2012;**116**(6):1379-88

67. Winkelman C, Johnson KD, Hejal R, et al. Examining the positive effects of exercise in intubated adults in ICU: a prospective repeated measures clinical study. Intensive & Critical Care Nursing 2012;**28**(6):307-18

68. Hildreth AN, Enniss T, Martin RS, et al. Surgical intensive care unit mobility is increased after institution of a computerized mobility order set and intensive care unit mobility protocol: a prospective cohort analysis. American Surgeon 2010;**76**(8):818-22

69. Needham DM, Korupolu R, Zanni JM, et al. Early physical medicine and rehabilitation for patients with acute respiratory failure: a quality improvement project. Archives of Physical Medicine & Rehabilitation 2010;**91**(4):536-42

70. Thomsen GE, Snow GL, Rodriguez L, Hopkins RO. Patients with respiratory failure increase ambulation after transfer to an intensive care unit where early activity is a priority. Critical Care Medicine 2008;**36**(4):1119-24

71. Booth K, Rivet J, Flici R, et al. Progressive Mobility Protocol Reduces Venous Thromboembolism Rate in Trauma Intensive Care Patients: A Quality Improvement Project. Journal of trauma nursing : the official journal of the Society of Trauma Nurses 2016;**23**(5):284-89

72. van Willigen Z, Collings N, Richardson D, Cusack R. Quality improvement: The delivery of true early mobilisation in an intensive care unit. BMJ Quality Improvement Reports 2016;**5**(1)

73. Borges RC, Carvalho CRF, Colombo AS, da Silva Borges MP, Soriano FG. Physical activity, muscle strength, and exercise capacity 3 months after severe sepsis and septic shock. Intensive Care Medicine 2015;**41**(8):1433-44

74. Wang YT, Haines TP, Ritchie P, et al. Early mobilization on continuous renal replacement therapy is safe and may improve filter life. Critical Care (London, England) 2014;**18**(4):R161

75. Gheith O, Al Otaibi T, Abdelhalim M, et al. Successful management of critical illness polyneuropathy and myopathy in renal transplant recipients. Experimental and Clinical Transplantation 2012;**10**(1):62-66

76. Bourdin G, Barbier J, Burle JF, et al. The feasibility of early physical activity in intensive care unit patients: a prospective observational one-center study. Respiratory Care 2010;**55**(4):400-7

77. Winkelman C. Investigating activity in hospitalized patients with chronic obstructive pulmonary disease: a pilot study. Heart & Lung 2010;**39**(4):319-30

78. Bailey P, Thomsen GE, Spuhler VJ, et al. Early activity is feasible and safe in respiratory failure patients. Crit Care Med 2007;**35**(1):139-45 doi: 10.1097/01.CCM.0000251130.69568.87[published Online First: Epub Date]|.

79. Zafiropoulos B, Alison JA, McCarren B. Physiological responses to the early mobilisation of the intubated, ventilated abdominal surgery patient. Australian Journal of Physiotherapy 2004;**50**(2):95-100

80. Malamud AL, Ricard PE. Feasibility of the Six-Minute Walk Test for Patients Who Have Cystic Fibrosis, Are Ambulatory, and Require Mechanical Ventilation Before Lung Transplantation. Physical therapy 2016;**96**(9):1468-76

81. Tadyanemhandu C, Manie S. Implementation of the physical function ICU test tool in a resource constrained intensive care unit to promote early mobilisation of critically ill patients- a feasibility study. Archives of Physiotherapy 2016;**6**:12

82. Moss M, Nordon-Craft A, Malone D, et al. A Randomized Trial of an Intensive Physical Therapy Program for Patients with Acute Respiratory Failure. Am J Respir Crit Care Med 2016;**193**(10):1101-10 doi: 10.1164/rccm.201505-1039OC[published Online First: Epub Date]|.

83. Morris PE, Berry MJ, Files DC, et al. Standardized Rehabilitation and Hospital Length of Stay Among Patients With Acute Respiratory Failure: A Randomized Clinical Trial. JAMA 2016;**315**(24):2694-702 doi: 10.1001/jama.2016.7201[published Online First: Epub Date]|.

84. Kayambu G, Boots R, Paratz J. Early physical rehabilitation in intensive care patients with sepsis syndromes-a randomised controlled trial. Physiotherapy (United Kingdom) 2015;**101**:eS735

85. Yosef-Brauner O, Adi N, Ben Shahar T, Yehezkel E, Carmeli E. Effect of physical therapy on muscle strength, respiratory muscles and functional parameters in patients with intensive care unit-acquired weakness. The clinical respiratory journal 2015;**9**(1):1-6

86. Brummel NE, Girard TD, Ely EW, et al. Feasibility and safety of early combined cognitive and physical therapy for critically ill medical and surgical patients: the Activity and Cognitive Therapy in ICU (ACT-ICU) trial. Intensive Care Medicine 2014;**40**(3):370-9

87. Denehy L, Skinner EH, Edbrooke L, et al. Exercise rehabilitation for patients with critical illness: a randomized controlled trial with 12 months of follow-up. Critical Care (London, England) 2013;**17**(4):R156

88. Chen YH, Lin HL, Hsiao HF, et al. Effects of exercise training on pulmonary mechanics and functional status in patients with prolonged mechanical ventilation. Respiratory Care 2012;**57**(5):727-34

89. Chen S, Su CL, Wu YT, et al. Physical training is beneficial to functional status and survival in patients with prolonged mechanical ventilation. Journal of the Formosan Medical Association 2011;**110**(9):572-9

90. Schweickert WD, Pohlman MC, Pohlman AS, et al. Early physical and occupational therapy in mechanically ventilated, critically ill patients: a randomised controlled trial. Lancet 2009;**373**(9678):1874-82 doi: 10.1016/S0140-6736(09)60658-9[published Online First: Epub Date]|.

91. Patman S, Sanderson D, Blackmore M. Physiotherapy following cardiac surgery: is it necessary during the intubation period? Australian Journal of Physiotherapy 2001;**47**(1):7-16

92. Nava S. Rehabilitation of patients admitted to a respiratory intensive care unit. Archives of Physical Medicine & Rehabilitation 1998;**79**(7):849-54

93. Dantas CM, Silva PF, Siqueira FH, et al. Influence of early mobilization on respiratory and peripheral muscle strength in critically ill patients. Revista Brasileira de Terapia Intensiva 2012;**24**(2):173-8

94. Parry SM, Berney S, Warrillow S, et al. Functional electrical stimulation with cycling in the critically ill: a pilot case-matched control study. Journal of Critical Care 2014;**29**(4):695.e1-7

95. Yang PH, Wang CS, Wang YC, et al. Outcome of physical therapy intervention on ventilator weaning and functional status. Kaohsiung Journal of Medical Sciences 2010;**26**(7):366-72

96. Malkoc M, Karadibak D, Yildirim Y. The effect of physiotherapy on ventilatory dependency and the length of stay in an intensive care unit. International Journal of Rehabilitation Research 2009;**32**(1):85-8

97. Hanekom S, Louw QA, Coetzee AR. Implementation of a protocol facilitates evidence-based physiotherapy practice in intensive care units. Physiotherapy 2013;**99**(2):139-45

98. Hodgson C, Bellomo R, Berney S, et al. Early mobilization and recovery in mechanically ventilated patients in the ICU: A bi-national, multi-centre, prospective cohort study. Critical Care 2015;**19 (1) (no pagination)**(81)

99. Sricharoenchai T, Parker AM, Zanni JM, Nelliot A, Dinglas VD, Needham DM. Safety of physical therapy interventions in critically ill patients: a single-center prospective evaluation of 1110 intensive care unit admissions. Journal of Critical Care 2014;**29**(3):395-400

100. Dinglas VD, Colantuoni E, Ciesla N, Mendez-Tellez PA, Shanholtz C, Needham DM. Occupational therapy for patients with acute lung injury: factors associated with time to first intervention in the intensive care unit. American Journal of Occupational Therapy 2013;**67**(3):355-62

101. Kho ME, Damluji A, Zanni JM, Needham DM. Feasibility and observed safety of interactive video games for physical rehabilitation in the intensive care unit: a case series. Journal of Critical Care 2012;**27**(2):219.e1-6

102. Zeppos L, Patman S, Berney S, Adsett JA, Bridson JM, Paratz JD. Physiotherapy in intensive care is safe: an observational study. Australian Journal of Physiotherapy 2007;**53**(4):279-83

103. Weissman C, Kemper M, Damask MC, Askanazi J, Hyman AI, Kinney JM. Effect of routine intensive care interactions on metabolic rate. Chest 1984;**86**(6):815-8

104. Weissman C, Kemper M, Elwyn DH, Askanazi J, Hyman AI, Kinney JM. The energy expenditure of the mechanically ventilated critically ill patient. An analysis. Chest 1986;**89**(2):254-9

105. Damluji A, Zanni JM, Mantheiy E, Colantuoni E, Kho ME, Needham DM. Safety and feasibility of femoral catheters during physical rehabilitation in the intensive care unit. Journal of Critical Care 2013;**28**(4):535.e9-15

106. Bartolo M, Bargellesi S, Castioni CA, et al. Early rehabilitation for severe acquired brain injury in intensive care unit: multicenter observational study. European journal of physical and rehabilitation medicine 2016;**52**(1):90-100

107. Jesus FS, Paim Dd.e M, Brito Jd.e O, et al. Mobility decline in patients hospitalized in an intensive care unit. Revista Brasileira de terapia intensiva 2016;**28**(2):114-19

108. Toonstra AL, Zanni JM, Sperati CJ, et al. Feasibility and safety of physical therapy during continuous renal replacement therapy in the intensive care unit. Annals of the American Thoracic Society 2016;**13**(5):699-704

109. Azuh O, Gammon H, Burmeister C, et al. Benefits of Early Active Mobility in the Medical Intensive Care Unit: A Pilot Study. American Journal of Medicine 2016;**129**(8):866-71.e1

110. Davis J, Crawford K, Wierman H, et al. Mobilization of ventilated older adults. Journal of Geriatric Physical Therapy 2013;**36**(4):162-8

111. Clini EM, Crisafulli E, Antoni FD, et al. Functional recovery following physical training in tracheotomized and chronically ventilated patients. Respiratory Care 2011;**56**(3):306-13

112. Nordon-Craft A, Schenkman M, Ridgeway K, Benson A, Moss M. Physical therapy management and patient outcomes following ICU-acquired weakness: a case series. Journal of Neurologic Physical Therapy 2011;**35**(3):133-40

113. Turner DA, Cheifetz IM, Rehder KJ, et al. Active rehabilitation and physical therapy during extracorporeal membrane oxygenation while awaiting lung transplantation: a practical approach. Critical Care Medicine 2011;**39**(12):2593-8

114. Ceriana P, Delmastro M, Rampulla C, Nava S. Demographics and clinical outcomes of patients admitted to a respiratory intensive care unit located in a rehabilitation center. Respiratory Care 2003;**48**(7):670-6

115. Sneyd J, Wang D, Edwards D, et al. Effect of physiotherapy on the auditory evoked response of paralysed sedated patients in the intensive care unit. Br J Anaesth 1992;**68**(4):349-51.

116. Hickmann CE, Castanares-Zapatero D, Bialais E, et al. Teamwork enables high level of early mobilization in critically ill patients. Annals of Intensive Care 2016;**6 (1) (no pagination)**(80)

117. Kamdar BB, Combs MP, Colantuoni E, et al. The association of sleep quality, delirium, and sedation status with daily participation in physical therapy in the ICU. Critical Care 2016;**20 (1) (no pagination)**(261)
